# Health problems among detainees in Switzerland: a study using the ICPC-2 classification

**DOI:** 10.1186/1471-2458-11-245

**Published:** 2011-04-19

**Authors:** Hans Wolff, Paul Sebo, Dagmar M Haller, Ariel Eytan, Gérard Niveau, Dominique Bertrand, Laurent Gétaz, Bernard Cerutti

**Affiliations:** 1 Department of community medicine and primary care, Geneva University Hospitals and University of Geneva, Switzerland; 2 Primary Care Practice, Thônex, Switzerland; 3 Department of psychiatry, Geneva University Hospitals and University of Geneva, Switzerland; 4 Institute of legal medicine; University of Geneva, Switzerland; 5 Faculty of Medicine, University of Geneva, Switzerland

**Keywords:** Primary care, prisoners, detainees, jail, ICPC, coding, access to care, prison health care

## Abstract

**Background:**

Little is known about the health status of prisoners in Switzerland. The aim of this study was to provide a detailed description of the health problems presented by detainees in Switzerland's largest remand prison.

**Methods:**

In this retrospective cross-sectional study we reviewed the health records of all detainees leaving Switzerland's largest remand prison in 2007. The health problems were coded using the International Classification for Primary Care (ICPC-2). Analyses were descriptive, stratified by gender.

**Results:**

A total of 2195 health records were reviewed. Mean age was 29.5 years (SD 9.5); 95% were male; 87.8% were migrants. Mean length of stay was 80 days (SD 160). Illicit drug use (40.2%) and mental health problems (32.6%) were frequent, but most of these detainees (57.6%) had more generic primary care problems, such as skin (27.0%), infectious diseases (23.5%), musculoskeletal (19.2%), injury related (18.3%), digestive (15.0%) or respiratory problems (14.0%). Furthermore, 7.9% reported exposure to violence during arrest by the police.

**Conclusion:**

Morbidity is high in this young, predominantly male population of detainees, in particular in relation to substance abuse. Other health problems more commonly seen in general practice are also frequent. These findings support the further development of coordinated primary care and mental health services within detention centers.

## Background

Prisoners are an underserved and vulnerable population. They frequently have had limited previous access to healthcare due to educational, social and economic disadvantage [1,2]. Prison has been identified as a significant opportunity to address the health needs of vulnerable groups. In particular, prison health services aim to reduce inequalities by providing primary care services that are similar in range and quality to those available in the community [3]. Addiction, psychiatric problems and infectious disease are recognized as important health problems in prison, their extent varies widely from one setting to another [1]. Belgian prisoners have been shown to make substantial use of primary care services during incarceration [4]. In US jails 36.9% of inmates in 2002 reported having a current medical problem but only 42% of them said that they had seen a health care professional about it. The most frequent specific health problems were dental problems (25%), arthritis (13%), followed by hypertension (11%) and asthma (10%). Furthermore, 13% of inmates reported being injured since admission [5].

The frequency and range of health problems encountered in prisons should shape the composition and competence profile of prison health care services. Yet little is known about the primary care needs of detainees in most European countries, and no detailed description is available for Switzerland. The aim of this study was thus to provide a detailed description of the health problems of detainees in Switzerland's largest remand prison.

## Methods

### Setting

In 2007, Switzerland had 115 institutions for 5715 prisoners of whom 29% were in pre-trial detention. This country had an average of 76 prisoners per 100'0000 residents, which is one of the lowest rates in the world [6]. Geneva however topped the national statistics with an average of 200 inmates per 100,000 residents [7]. The majority of detainees in Swiss jails were male (93.6%) and of foreign origin (81.4%) [6]. Furthermore, the canton of Geneva has the highest proportion of foreigners (38.3%) among its general resident population [8].

The study took place in Switzerland's largest remand prison, situated in Geneva and built in 1977. Initially conceived to receive 270 prisoners, this prison is overcrowded with a mean occupation rate of 169% in 2007. At the time the study took place, 10 to 20% of detainees were sentenced prisoners waiting to be transferred to another institution. The medical unit attached to Geneva University Hospitals is completely independent of the prison administration. It offers a low-threshold primary care approach to health care and employs 37 health professionals, including general practitioners, nurses, psychiatrists, psychologists and dentists. The facility operates 24 h/day with a nurse present at all times. All detainees admitted to the facility are submitted to a health assessment by primary health care nurses within the first 8 hours of their admission. This evaluation acts as triage to identify any health problems requiring medical attention, such as allergies, injuries, breathing problems, mental health problems including suicidal ideas, addiction, regular medical treatment, suspicion of tuberculosis or allegations of violence during arrest. The nurse evaluation is also an introduction to the facility's health service. When necessary, nurses refer detainees immediately to a primary care physician. At any time, inmates can ask for a medical consultation and are then addressed to a primary care physician. Referrals to the psychiatric team occur via the primary care physician.

### Instrument and design

This retrospective, cross-sectional study assessed the health problems of all detainees leaving the facility between January 1st and December 31st in 2007. All health records (nurse evaluation forms and medical files) were reviewed and coded using the French version of the international classification of primary care, second edition (ICPC-2) [9,10]. This coding is particularly adapted to primary care where complaints do not always lead to a specific diagnosis. The questionnaire (additional file 1) grouped ICPC-2 diagnoses within six categories created to reflect the clinical reality of prison medicine: 1. symptoms without precise diagnosis, 2. substance abuse or self-harm, 3. infections, 4. general internal medicine, 5. psychiatry and 6. injuries.

Daily use of at least one cigarette defined tobacco use. The first three questions of the Alcohol Use Disorders Identification Test (AUDIT) were used to assess alcohol misuse defined as excessive drinking including heavy drinking, binge drinking or both, or alcohol abuse or dependence. The standard 10-item AUDIT [11] has been tested and validated in primary care, but its length precludes its use in jail settings. The derived three-item AUDIT-C has shown good screening performance for alcohol use disorders and risky drinking, and is now considered a reliable alternative to the standard AUDIT score [12,13]. All detainees were asked about illicit substance use during the initial nurse evaluation. Prisoners referred to the primary care physician were systematically screened again for substance use during the first consultation. Active cocaine, respectively heroin use was classified as positive if used during the last 30 days before entering the prison. Previous cocaine, respectively heroin use was recorded if prisoners reported lifetime but no active use. Regular use of cannabis or benzodiazepine (more than once a week, without medical prescription) was recorded if the prisoner reported use during the last 30 days before admission to the prison. Cocaine, heroin and cannabis use were grouped as "illicit drug use". All detainees were asked about exposure to violence during arrest in the initial nurse evaluation and referred to the primary care physician if such exposure was reported. Socio-demographic data were age, gender, nationality (as a proxy for origin). Length of stay was recorded as it could be correlated with morbidity. Detainees who stay longer in prison have a higher probability of developing health problems and being in contact with health services.

The methodology and the coding instrument were pre-tested over a three-month period (October 2006 to December 2006). Approximately 400 files were analyzed during this pre-test. It allowed improvements to be made to the research procedure. A codebook was established to harmonize the use the ICPC-2 coding system within the research team. All health records were then coded following this procedure. One coder (DB) reviewed all the files and followed strict coding rules established by the research team at the initiation of the study. Coding doubts were discussed and resolved during regular meetings both with another coder in a different detention setting (DMH) and the entire research team. All data were recorded anonymously. Our focus was on somatic health problems and substance abuse. A more detailed description of specific psychiatric health problems are presented in a separate article [14].

### Statistical analysis and ethical approval

Descriptive statistics were computed for demographic characteristics. The frequencies of ICPC-2 coded health problems were computed with 95% confidence intervals. Unless specified, we used Chi square tests to explore possible associations between commonly occurring health problems and patient characteristics. Age and sex-adjusted odds ratios for the association between origin and health problems were obtained with logistic regression models. Statistical analyses were done with S-Plus 7.0 Enterprise Developer and SPSS 15.0. The research project was approved by the Ethics Committee of Geneva University Hospitals.

## Results

### Sociodemographic characteristics

Of 2195 prisoners leaving the prison during 2007, 1510 (68.8%) had a primary care consultation during their stay and 685 (31.2%) only had an initial health assessment by the nurse. Hundred-fifteen different nationalities were represented and 92.8% detainees with at least one medical consultation were of foreign origin. Main regions of origin were Western Europe (28.9%), followed by North Africa and Middle East (27.5%) and Sub-Saharan Africa (20.1%). More details are shown in table 1. Length of stay was short as 27% stayed less than one week and 78% less than three months in the prison.

**Table 1:** Socio-demographic characteristics of 2195 detainees in a remand prison in Geneva, Switzerland, 2007

Variable	Detainees with at least one medical consultation N = 1510	Detainees without medical consultation N = 685	All detainees N = 2195
Age (years (SD, range))	30.1 (9.8, 18-82)	28.0 (8.8, 18-71)*	29.5 (9.5, 18-82)*

Sex male (n)	95% (1434)	95% (653)	95% (2087)
Length of stay in prison (days (SD, range))	98 (179, 1-2635)**	37 (89, 1-1010)***	80 (160, 1-2635)****

Origin (continent and 5 most frequent countries):			

Western Europe§§	27.4% (413)	32.7% (208)	28.9% (621)
France	8.9% (135)	9.4% (60)	9.1% (195)
Switzerland	7.7% (116)	8.6% (61)	8.2% (177)
Italy	2.3% (35)	2.4% (15)	2.3% (50)
Portugal	2.1% (31)	2.7% (17)	2.2% (48)
Spain	2.1% (31)	1.1% (7)	1.8% (38)

Eastern Europe	14.6% (221)	22.0% (140)	16.8% (361)
Albania	5.5% (83)	13.8% (88)	8.0% (171)
Romania	2.0% (30)	4.6% (29)	2.7% (59)
Russia	2.7% (40)	0.6% (4)	2.1% (44)
Kosovo	1.7% (25)	0.9% (6)	1.4% (31)
ex-Yugoslavia§	1.6% (22)	1.4% (9)	1.4% (31)

North Africa and Middle East	30.6% (461)	20.3% (129)	27.5% (590)
Algeria	15.8% (239)	8.6% (55)	13.7% (294)
Palestine	5.8% (88)	3.1% (20)	5.0% (108)
Iraq	3.6% (55)	2.2% (14)	3.2% (69)
Morocco	3.6% (54)	1.7% (11)	3.0% (65)
Tunisia	1.7% (26)	2.5% (16)	2.0% (42)

Sub-Saharan Africa	20.9% (315)	18.2% (116)	20.1% (432)
Guinea	5.2% (78)	5.3% (34)	5.2% (112)
Sierra Leone	1.9% (29)	1.6% (10)	1.8% (39)
Ivory Coast	1.5% (23)	1.6% (10)	1.5% (33)
Congo	1.1% (16)	1.4% (9)	1.2% (25)
Mali	1.0% (15)	1.3% (8)	1.1% (23)

Asia	3.8% (57)	2.2% (14)	3.3% (71)
Georgia	2.7% (41)	1.1% (7)	2.2% (48)
Vietnam	0.2% (3)	0.5% (1)	0.2% (4)
Philippines	0.2% (3)	-	0.1% (3)
Azerbaijan	0.1% (2)	-	0.1% (2)
Uzbekistan	0.1% (2)	-	0.1% (2)

America	2.8% (42)	4.4% (28)	3.3% (70)
Chile	0.7% (11)	0.6% (4)	0.7% (15)
Brazil	0.7% (10)	0.8% (5)	0.7% (15)
Peru	0.3% (4)	0.6% (4)	0.4% (8)
Colombia	0.1% (2)	0.9% (6)	0.4% (8)
Jamaica	0.3% (4)	0.2% (1)	0.2% (5)

### ICPC coded health problems

**Figure 1:**
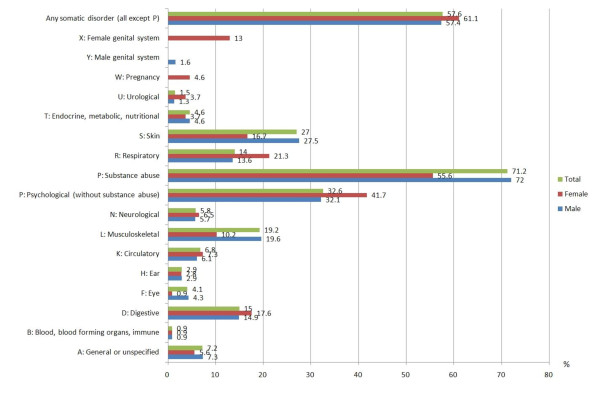
**ICPC-2 coded health problems in 2195 of detainees in a remand prison in Geneva, Switzerland, 2007**.

Figure 1 describes the ICPC coded health problems identified in 2195 health records. Morbidity was high; both somatic (57.6%) and mental (32.6%) health problems were highly prevalent. There were no gender differences in relation to the percentage of somatic health problems which were observed in female (61.1%; 95%CI 51.9-70.3) and male (57.4%; 95%CI 55.3-59.5) of detainees (p = 0.45). Major somatic health problems were: 1. skin (27.0%), 2. musculoskeletal (19.2%), 3. digestive (15.0%) and 4. respiratory (14.0%) disorders. Somatic disorders were slightly more frequent in those 29 years (median) or older (64% vs. 53%, p < 0.0001). Mean number of identified health problems were 2.4 (SD 1.8). Those who stayed less than 1 month had a mean of 2.0 (SD 1.3), those who stayed 3 to 6 months a mean of 3.2 (SD 2.0) and those who stayed more than 6 months a mean of 4.1 (SD 2.6) identified health problems.

### Symptoms or complaints without precise diagnosis

**Figure 2:**
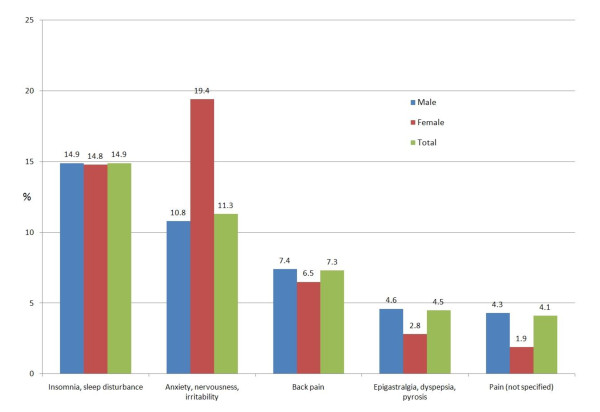
Symptoms coded without precise diagnosis (42.2%, 95%CI 40.1-44.3)

Symptoms or complaints without precise diagnosis, such as insomnia, back pain or feeling anxious, were present in 926 detainees (42.2%). Their prevalence is presented in figure 2 and table 2.

**Table 2:** Frequencies of ICPC-2 coded health problems of 2195 medical files of detainees in a remand prison in Geneva, Switzerland, 2007

Category (common examples)	Males		Females		All detainees		Prevalence data general population
	N = 2087		N = 108		N = 2195		
	N	% (95% CI)	N	42.6 (33.3-51.9)	N	% (95% CI)	%

A. Symptoms without precise diagnosis	880	42.2 (40.0-44.3)	46	14.8 (8.1-21.5)	926	42.2 (40.1-44.3)	

Insomnia, sleep disturbance	310	14.9 (13.3-16.4)	16	19.4 (12.0-26.9)	326	14.9 (13.4-16.3)	4.1-6.9 [26]*

Anxiety, nervousness, irritability, anger	226	10.8 (9.5-12.2)	21	6.5 (1.8-11.1)	247	11.3 (9.9-12.6)	

Back pain	154	7.4 (6.3-8.5)	7	6.5 (1.8-11.1)	161	7.3 (6.2-8.4)	5.2-8.8 [26]*

Epigastralgia, dyspepsia, pyrosis	96	4.6 (3.7-5.5)	3	2.8 (0.0-5.9)	99	4.5 (3.6-5.4)	3.7-4.4 [26]*

Pain (not specified)	89	4.3 (3.4-5.1)	2	1.9 (0.0-4.4)	91	4.1 (3.3-5.0)	

							

B. Infectious diseases	485	23.2 (21.4-25.1)	31	28.7 (20.2-37.2)	516	23.5 (21.7-25.3)	

Upper airways infection	155	7.4 (6.3-8.6)	13	12.0 (5.9-18.2)	168	7.7 (6.5-8.8)	

Mycosis (genital excepted)	138	6.6 (5.6-7.8)	2	1.8 (0.0-4.3)	140	6.4 (4.0-5.8)	

Folliculitis, Furunculosis	59	2.8 (2.1-3.5)	1	0.9 (0.0-2.7)	60	2.7 (2.1-3.4)	

Other prison relevant infections:							

Pediculosis, scabies	19	0.9 (0.5-1.3)	0		19	0.9 (0.5-1.3)	

Hepatitis B (chronic active)	22	1.1 (0.6-1.5)	0	7.4 (2.5-12.3)	22	1.0 (0.6-1.4)	0.2 [36]

Hepatitis C	118	5.7 (4.7-6.6)	8	7.4 (2.5-12.3)	126	5.7 (4.8-6.7)	0.7-1.0 [20]

HIV, AIDS	19	0.9 (0.5-1.3)	5	2.8 (0-5.9)	22	1.0 (0.6-1.4)	0.3 [36]

Tuberculosis (active)	5	0.2 (0.0-0.4)	3		5	0.2 (0.0-0.6)	0.006 [19]

							

C. General internal medicine	319	29.7 (27.7-31.6)	37	34.3 (25.3-43.2)	656	29.9 (28.0-31.8)	

Xerosis with pruritus	143	6.9 (5.8-7.9)	2	1.9 (0.0-4.4)	145	6.6 (5.6-7.6)	

Asthma	67	3.2 (2.5-4.1)	3	2.8 (0.0-5.9)	70	3.2 (2.5-3.9)	2.5 [25]

Acne	51	2.4 (1.8-3.1)	4	3.7 (0.1-7.3)	55	2.5 (1.9-3.2)	

Eye disease (without conjunctivitis)	54	2.6 (1.9-3.3)	1	0.9 (0.0-2.7)	55	2.5 (1.9-3.2)	

Hypertension (arterial)	47	2.3 (1.6-2.9)	6	5.6 (1.2-9.9)	53	2.4 (1.8-3.1)	2.5-7.6 [24]*

Allergy	45	2.2 (1.5-2.8)	5	4.6 (0.7-8.6)	50	2.3 (1.7-2.9)	

Eczema	47	2.3 (1.6-2.9)	2	1.9 (0.0-4.4)	49	2.2 (1.6-2.9)	

Dyslipidemia, hypercholesterolemia	42	2.0 (1.4-2.6)	3	2.8 (0.0-5.9)	45	2.1 (1.5-2.6)	1.1-6.3 [24]*

Hemorrhoids	39	1.9 (1.3-2.4)	1	0.9 (0.0-2.7)	40	1.8 (1.3-2.4)	

Migraine, tension headache	32	1.5 (1.0-2.1)	5	4.6 (0.7-8.6)	37	1.7 (1.1-2.2)	7.7-9.3 [26]

							

D. Injuries	392	18.8 (17.1-20.5)	9	8.3 (3.1-13.5)	401	18.3 (16.7-19.9)	13.7-25.7 [24]*

Alleged victim of violence	166	8.0 (6.8-9.1)	7	6.5 (1.7-11.1)	173	7.9 (6.8-9.0)	

Contusion (without skin lesion)	141	6.8 (5.7-7.8)	7	6.5 (1.8-11.1)	148	6.7 (5.7-7.8)	

Contusion (with skin lesion)	124	5.9 (4.9-7.0)	3	2.8 (0.0-5.9)	127	5.8 (4.8-6.8)	

Sprained ankle	41	2.0 (1.4-2.6)	1	0.9 (0.0-2.7)	42	1.9 (1.3-2.5)	

Periarticular lesion	33	1.6 (1.0-2.1)	0		33	1.5 (1.0-2.0)	

Self harm	98	4.7 (3.8-5.6)	2	1.9 (0.0-4.4)	100	4.6 (3.7-5.4)	

During imprisonment	50	2.4 (1.7-3.1)	1	0.9 (0.0-2.7)	51	2.3 (1.7-3.0)	
Previous	57	2.7 (2.0-3.4)	1	0.9 (0.0-2.7)	58	2.6 (2.0-3.3)	

*adults < 49 years

### Substance abuse

**Figure 3:**
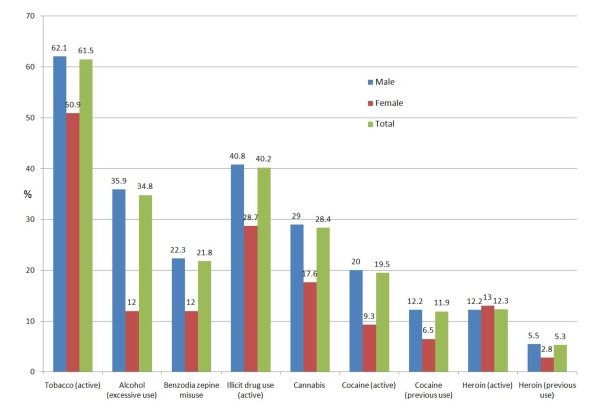
Health problems coded as substance abuse (71.2%; 95%CI 69.3-73.1)

Substance abuse was frequent and observed in 1562 detainees (71.2%): 61.5% smoked tobacco and 34.8% reported excessive alcohol consumption. Forty percent used at least one illicit drug (heroin, cocaine or cannabis) in the 30 days before admission. Lifetime consumption of cocaine or heroin was 33.8%. All substances were more frequently used by men compared to women (details are presented in figure 3 and table 3).

**Table 3:** Frequencies of ICPC-2 coded health problems (Substance abuse, self harm and psychiatric disorders) of 2195 medical files of detainees in a remand prison in Geneva, Switzerland, 2007

Category (common examples)	Males		Females		All detainees		Prevalence data: General population
	N = 2087		N = 108		N = 2195		
					N	% (95% CI)	

E. Substance abuse							

Substance abuse (licit)	1502	72.0 (70.0-73.9)	60	55.6 (46.2-64.9)	1562	71.2 (69.3-73.1)	

Tobacco (active)	1296	62.1 (60.0-64.2)	55	50.9 (41.5-60.4)	1351	61.5 (59.5-63.6)	29.3-37.2 [24]*

Alcohol misuse	750	35.9 (33.9-38.0)	13	12.0 (5.9-18.2)	763	34.8 (32.8-36.8)	3.9-4.8 [24]*

Benzodiazepine (not medically prescribed)	465	22.3 (20.5-24.1)	13	12.0 (5.9-18.2)	478	21.8 (20.1-23.5)	

Illicit drug use (active):	852	40.8 (38.7-42.9)	31	28.7 (20.2-37.2)	883	40.2 (38.2-42.3)	

Cannabis	605	29.0 (27.0-30.9)	19	17.6 (10.4-24.8)	624	28.4 (26.5-30.3)	9.3 [24]

Cocaine	418	20.0 (18.3-21.7)	10	9.3 (3.8-14.7)	428	19.5 (17.8-21.2)	2.8 [21]**

Heroine	255	12.2 (10.8-13.6)	14	13.0 (6.6-19.3)	269	12.3 (10.9-13.6)	0.7 [21]**

Illicit drug use (lifetime):	720	34.5 (32.5-36.5)	21	19.4 (12.0-26.9)	741	33.8 (31.8-35.7)	

Cocaine	673	32.2 (30.2-34.3)	17	15.7 (8.9-22.6)	690	31.4 (29.5-33.4)	2.8 [21]*

Heroine	369	17.7 (16.0-19.3)	17	15.7 (8.9-22.6)	386	17.6 (16.0-19.2)	0.7 [21]**

							

F. Psychiatry	332	15.9 (14.3-17.5)	27	25.0 (16.8-17.9)	359	16.4 (14.8-17.9)	

Depression	145	6.9 (5.9-8.0)	18	16.7 (9.6-23.7)	163	7.4 (6.3-8.5)	2-9% major depression [29]

Personality disorder	111	5.3 (4.4-6.3)	9	8.3 (3.1-13.5)	120	5.5 (4.5-6.4)	1-3% antisocial personality disorder [29]

Adjustment disorder	90	4.3 (3.4-5.2)	8	7.4 (2.5-12.3)	98	4.5 (3.6-5.3)	5-20% in individuals seeking outpatient mental health treatment [29]

Post Traumatic Stress Disorder	22	1.1 (0.6-1.5)	0		22	1.0 (0.6-1.4)	1-14% [29]

Psychosis (schizophrenia, delirium)	17	0.8 (0.4-1.2)	4	3.7 (0.0-7.3)	21	1.0 (0.5-1.4)	0.2-2% schizophrenia [29]

Bipolar disorder	2	0.1 (0.0-0.2)	0		2	0.1 (0.0-0.2)	0.4-1.6 [29]

*adults < 49 years, ** active and previous

### Infectious diseases

**Figure 4:**
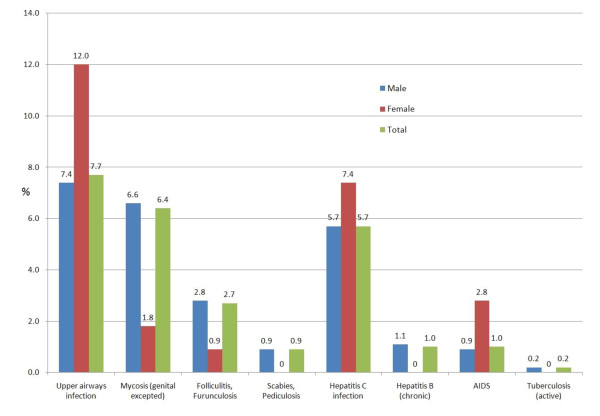
Health problems coded as infectious diseases (23.5%; 95%CI 21.7-25.3)

Infectious diseases were found in 23.5%. As shown in figure 4, upper airway infection was the most frequent infection, followed by fungal infections. Hepatitis C infection was found in 5.7% of detainees and among 15.4% (95%CI 12.8-18.0) of those who used either heroin or cocaine on admission. A fifth of Georgians, who also showed high prevalence rates of illicit drug (see below), were infected by HCV (20.8% CI95% 9.3-32.3). Only 0.8% (95%CI 0.4-1.3) of non-users of illicit drugs had hepatitis C infection. Chronic Hepatitis B was identified in 1% and HIV infection in 1% of the inmates.

### Health problems grouped as "general internal medicine"

**Figure 5:**
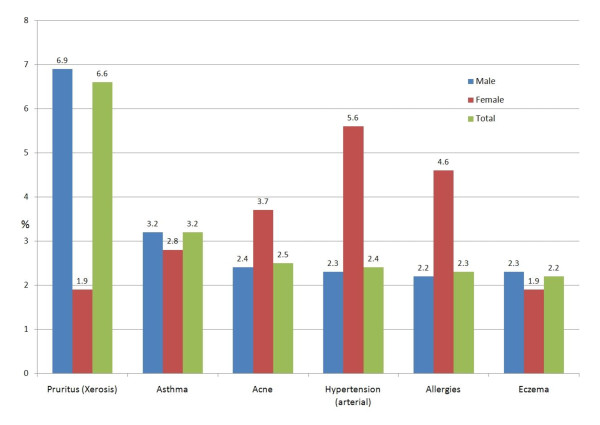
Health problems coded as general internal medicine (29.9%; 95%CI 28.0-31.8)

Thirty percent had health problems grouped in this category (figure 5, table 2). Skin problems, such as xerosis with pruritus, acne or eczema were predominant in this group. Asthma was the second most frequent health problem, observed in 3.2% of men and 2.8% of women. Diabetes was identified in 1.3% (CI95% 08-18) of male and in no female detainee.

### Psychiatric disorders (excluding substance abuse)

**Figure 6:**
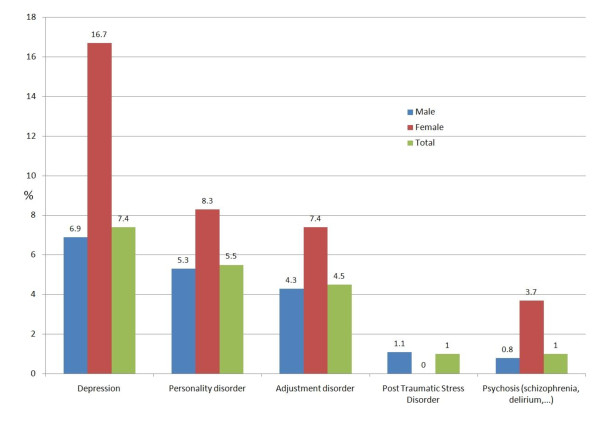
Health problems coded as psychiatry (16.4%; 95%CI 14.8-17.9)

Excluding substance abuse, psychiatric problems were found in 15.9% of men and 25% of women (figure 6). Depression was observed more than twice as frequently in women (16.7%) than in men (6.9%). Other frequently observed disorders were personality (5.5%) and adjustment disorders (4.5%).

### Exposure to violence and injury

**Figure 7:**
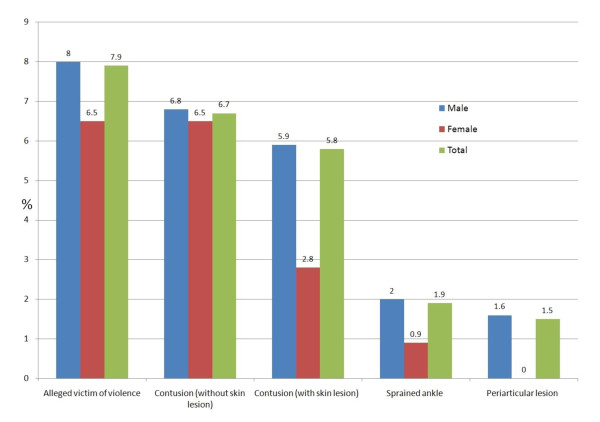
Health problems coded as injuries (18.3%; 95%CI 16.7-19.9)

Injuries occurred in 18.3% of detainees and were more than twice as frequent in men (18.8%) compared to women (8.3%) (figure 7). Nearly one in ten detainees alleged being a victim of violence during arrest; 90% of these blaming the police. No significant gender differences were found but alleged exposure to violence by the authorities was more frequently declared by younger inmates (≤28 years) compared to older detainees (>28 years) (9.4% vs 6.1%, p = 0.0037).

Self harm (drug overdose, self-mutilation and swallowing of foreign bodies) was observed in 1.9% of women and 4.7% of men. No death occurred in the institution during 2007.

### Origin-related health problems

Compared to Western Europeans who served as the reference group, sub-Saharan Africans had a higher burden of infectious diseases (OR 1.62, CI95% 1.21-2.17), and detainees from North-Africa/Middle-East more psychiatric problems (OR 1.47, CI95% (1.09-1.98). Other statistical significant differences were observed only for substance abuse related problems: Asians had a higher prevalence of heroin use (OR 2.06, CI95%1.14-3.73). In particular Georgians had high prevalence rates of illicit drug use (56.2%, CI95% 42.2-70.3). A quarter of them used heroin (27.1%; CI95% 14.5-39.7) and 31.2% cocaine (CI95% 18.1-44.4). Detainees from North-Africa/Middle-East were more likely to use benzodiazepines (OR 1.81; CI95% 1.38-2.37), cocaine (OR 1.5, CI95% 1.13-2.01) and alcohol (OR 1.48; CI95% 1.16-1.87). Lower rates were found for Eastern Europeans for cannabis (OR 0.67; CI95% 0.49-0.92) and alcohol misuse (OR 0.69; CI95% 0.51-0.92).

## Discussion

This study is the first to detail the wide range of health problems among detainees in a Swiss remand prison. Illicit drug use (40.2%) and mental health problems (32.6%) were frequent in this mainly migrant population, but most of these detainees (57.6%) required care for common primary care problems, such as skin (27.0%), infectious diseases (23.5%), musculoskeletal (19.2%), injury related (18.3%), digestive (15.0%) or respiratory problems (14.0%). Until recently Switzerland lacked national comparative data concerning health problems in primary care. With the advent of electronic medical records this is slowly changing. A recent publication provides data about ICPC-2 coded electronic medical records of 24 Swiss GPs concerning 29'398 doctor-patient encounters. Main reasons for encounter were musculoskeletal (21%), circulatory (19%), respiratory (9%) and endocrine, metabolic and nutritional disorders (8%) [15]. Prevalence data in our study were different for most health problems (with the exception of musculoskeletal disorders). This adds value to our findings in highlighting the extent to which primary care services in prison may differ from those provided to the general population. Our findings are consistent with findings in Britain [16] and Belgium [4], where detainees consulted 3 to 3.8 times more frequently compared to the age-and sex-adjusted general population. Main ICPC-2 code groups in the Belgium study were the same as in ours. Nearly 70% of detainees in our study had consultations with a primary care physician. The high need for primary care services in prison can be explained by the accumulation of negative social determinants of health which contributes to a high burden of disease [2,4,17] but also by improved access to health care services in prison as well as the prison culture. For example, self-medication is usually forbidden in prison. Prisoners therefore have few opportunities to resort to self-care and are more likely to request medical help even for simple complaints [16].

Publications concerning US jail inmates report the following most frequent medical problems: arthritis (13-20%), hypertension (11-21%), asthma (10-24%), heart problems (5.9-11%), hepatitis (2.6-10%) and diabetes (2.7-6.5%) [5,18] Gender differences were observed with higher burden of chronic medical disorders, psychiatric disorders and drug dependence in women compared to men [18]. Our study identified higher burden of psychiatric disorders in women, an equal burden of somatic health problems in both groups and a higher burden of drug dependence in men. Cardiovascular risk factors such as hypertension (3.5%) and diabetes (1.2%) appear to be less frequent in the population involved in our study. As blood pressure was checked among all prisoners during the initial nurse evaluation, measurement bias is unlikely to explain the low proportion of hypertension in our study. In addition our findings in relation to hypertension and diabetes are well in line with population data in Switzerland, suggesting that, with the exception of tobacco cessation counseling, these detainees may have little need for cardiovascular preventive care.

Our study identified prevalence rates ≥10 times those found in the general population for tuberculosis (0.2% vs. 0.006% [19]), HCV infection (5.7% vs. 0.7-1% [20] and heroin use (12.3% vs. 0.7%[21]). Worldwide, TB-notification rates are found to be 14.9 times higher in prisoners compared to civilians, in Western Europe TB-notification rates are 8.1-times higher [22]. The high rates of infectious diseases and particularly of tuberculosis are related to risk factors which aggregate in prison: low socioeconomic status, intravenous drug use, homelessness, lack of access community based health care as well as the origin of high TB-incidence countries [22,23]. Our findings highlight the vulnerability of detainees to TB and other infectious diseases and stress the need for effective screening and containment measures in prison [22]. The frequency of common health problems such as dyslipidemia, back pain, or epigastralgia was well in line with that observed in the general Swiss population [24-26]. Asthma was less frequently reported than in prison settings in the US, UK or Australia [18,23], but in accordance with general population data of Switzerland [27].

Most psychiatric disorders were observed more frequently than in the general population. However, the proportion of detainees with psychiatric disorders was low compared to available data from the literature where prevalence rates of up to 65% for personality disorder (including 47% with antisocial personality) were found [23,28]. Several reasons may explain these findings. First, the health problems were identified by general practitioners and not by specialists. The ICPC-2 tool favors the identification of symptoms and complaints where diagnoses are not available and is particularly adapted for ambulatory settings in primary care. Second, we did not systematically use a diagnostic screening interview which explains why certain diagnoses were less prevalent in our study than usually described in the literature. This appears to be particularly true for long lasting problems such as personality disorders compared with acutely symptomatic mental disorders. Third, remand detainees differ from the local Swiss population regarding several socio-demographic characteristics, all of which are relevant for the prevalence, course and outcome of most mental disorders. More specifically, compared with the general Swiss population, detainees included in our study were younger, more often foreign born and with lower socioeconomic status. However, in order to allow for a broad comparison, ranges of prevalence mentioned in the DSM-IV [29] for specific disorders are presented in Table 3.

Our findings in relation to the high rates of heroin, cocaine and benzodiazepine use were well in range with those from previous studies, both in the US and in Europe [30-33]. That 40.2% detainees used at least one illicit drug at entry highlights the need for general practitioners (GP) working in detention to be well trained in addiction medicine. Offering adequate care to detainees who suffer from drug addiction is an important priority. It is particularly important to implement harm reduction strategies, such as opioid substitution treatment which has been shown to be a powerful tool to: 1. decrease the level of injecting, 2. prevent the transmission of blood-borne viruses, 3. prevent drug-related prison violence and crime following release as well as recidivism [30,34]. Furthermore, excessive use of alcohol (34.8%) and tobacco (61.5%) requires particular attention. Smoking rates were more than twice as high as in the general population in Switzerland where 32% of men and 23.8% of women smoked in 2007 [35]. Smoking is highly problematic in confined institutions as exposure to passive smoking is almost inevitable for everyone: inmates, prison officers and health professionals.

A challenge for the GP working in prison is continuity of care. Disease management requires good coordination with different health services before and after imprisonment. This is particularly important for medical management of infectious diseases such as hepatitis which is more prevalent in prison compared to the general population [36]. Fifty-six percent of iv drug users in Switzerland are infected with hepatitis C virus (HCV) [37]. Our study identified hepatitis C in 15.4% of those who reported using either heroin or cocaine on admission compared to 0.8% who denied use of illicit drugs. Vescio showed in a meta-analysis that HCV sero-prevalence in prison is closely related to the proportion of iv drug users in prison who are found to have a 24 times higher risk of HCV infection [38]. We identified highest sex- and age-adjusted opiate-consumption rates in Asians. These findings are concordant with international prevalence studies which confirm high rates of opiate and intravenous drug use in Asia. This region represents almost one-quarter of people who inject drugs worldwide [39]. In particular Georgia has one of the highest intravenous drug use rates worldwide (4.2% of 15-64 year olds), compared to Switzerland where 0.65% of 15-64 year olds inject drugs [40]. Georgians in our sample had high prevalence rates of HCV infection which were four times higher than the average (20.8% vs 5.7%). High rates of hepatitis C in this sample, even in the absence of a systematic screening policy, confirm the need to develop such a policy in our setting, and country of origin could serve as a rough decision-making criterion for ordering additional health screening. Imprisonment has to be considered as an opportunity to provide medical care and preventive measures to this hard-to-reach population. Furthermore, hepatitis C treatment is proven cost-effective in prison settings [41] but can only be effective if the prison health services and particularly the GP is able to achieve continuity of care.

Following systematic screening upon admission, alleged violence from the authorities was reported by 8% of detainees. Our health service has set-up a system for reporting these alleged violent events to a state ombudsman in order to favor a reduction in unethical behavior in law enforcement state services.

In the absence of systematic screening policies in our institution, the identification of problems such as STI, AIDS, hepatitis and psychiatric illnesses was based on clinical evaluation and/or patient requests for tests. This may have led to underestimations of the frequency of these health problems. Yet, our primary aim was not to provide precise prevalence data but to offer data on frequently encountered health problems in order to inform prison healthcare services development. Even if our findings provide conservative estimates of prevalence rates, they underline the high morbidity in this population in particular in relation to addiction and infectious diseases but also in relation to more common health problems seen in general practice. That all detainees underwent a health evaluation and were systematically referred to the physician when a medical problem was identified adds strength to the notion that, despite its limitations, our study provides a comprehensive picture of the health status of this population.

Another limitation was that data were extracted retrospectively by only one coder, a primary care physician who had not necessarily been involved in the care of each of the patients. Errors in coding, however, were minimized by setting strict rules for coding at the initiation of the study. Doubts were discussed and resolved in regular meetings with the research team. Finally, we studied a single facility in one country. Thus our findings may not necessarily be generaliseable to other detention centers in other countries. Yet we hypothesize that the health profile described here would be comparable in other detention centers where the sociodemographic profile corresponds to that described here. The study population was similar to that of other pre-trial prisons in Switzerland, which are also characterized by high proportions of migrants (81.4%) and males (94%) [6]. Factors contributing to the high rates of incarceration in migrants include high rates of illegal migration throughout Europe, the lower socio-economic status of this group and the fact that they are less likely to be granted a bail sentence. The high proportion of foreigners in Swiss jails underlines the need for culturally-sensitive approaches and non-stigmatizing attitudes towards this vulnerable population.

Strengths of the study include the large sample size including inmates of both genders. Information was gathered on all inmates present in the detention facility over an entire year. The use of the ICPC-2 coding system allowed us to capture the full range of health needs for which these inmates could benefit from primary care services upon admission and during their incarceration.

## Conclusions

The wide range of health problems that were identified in this prison population highlights the need for GP working in prison to acquire skills in many domains, including general internal medicine, addiction medicine, psychiatry and language- and culturally-appropriate communication with patients. Prison health services and prison authorities should carefully assess the possibilities for strengthening self-care options in prison in order to reduce the burden of self-limited illnesses on primary care services. For many inmates, contact with the prison health service is their first ever opportunity to meet a health professional. Addressing the health needs of these prisoners through well coordinated psychiatric and primary care services offers an opportunity to reach-out to this hard-to-reach vulnerable population in the interest of their individual health and of public health.

## Abbreviations

ICPC: International Classification of Primary Care; GP: General Practitioner; OST: Opioid Substitution Treatment; CI: confidence interval; OR: odds ratio; SD: standard deviation.

## Competing interests

The authors declare that they have no competing interests.

## Authors' contributions

HW participated in the study design and its coordination, the interpretation of the data, and drafted the manuscript. PS, DMH and GN participated in the study design, its coordination, the interpretation of the data, and gave critical contribution to the manuscript. BC participated in the study design, performed the statistical analysis, participated in the interpretation of the data and gave critical contribution to the manuscript. AE participated in the interpretation of the data, and gave critical contribution to the manuscript. DB participated in the study design, its coordination, data collection, the interpretation of the data, and gave critical contribution to the manuscript. LG participated in the literature review, interpretation of the data, and gave critical contribution to the manuscript. All authors read and approved the final manuscript.

## Pre-publication history

The pre-publication history for this paper can be accessed here:

http://www.biomedcentral.com/1471-2458/11/245/prepub

## Supplementary Material

Additional file 1 **Questionnaire ICPC-2, prison Champ-Dollon, Geneva, Switzerland 2007**. Click here for file
