# Supplementary material for: Health problems among detainees in Switzerland: a study using the ICPC-2 classification
Source: BMC Public Health. 2011 Apr 19;11:245. doi: 10.1186/1471-2458-11-245 (PMC3108317; doi:10.1186/1471-2458-11-245)
Supplement: Additional file 1 — Questionnaire ICPC-2, prison Champ-Dollon, Geneva, Switzerland 2007. [file 1471-2458-11-245-S1.DOC]

**Additional file 1 : Questionnaire ICPC-2, prison Champ-Dollon, Geneva, Switzerland 2007**

| **Socio-demographic characteristics:** | | | |
| --- | --- | --- | --- |
| Age | | | |
| Nationality | | | |
| Sex | | | |
| Date of departure | | | |
|  | | | |
| **A) Symptoms/complaints without diagnosis** | | | |
|  | Adenopathy, lymph gland enlarged/painful B02 |  | Neck/back/low back pain, without radiating pain L01, L02, L03, L83, L84 |
|  | Anorexia, loss of appetite T03 |  | Neck/back/low back pain, with radiating pain L83, L86 |
|  | Asthenia, weakness, tiredness A04 |  | Pain, other A29 |
|  | Bloating, flatulence, gas, belching D08 |  | Rectal/anal pain D04 |
|  | Claudication, ataxia, gait disorder N29 |  | Palpitation K04 |
|  | Constipation, hard stool D12 |  | Paresthesia, tingling, sensation disturbance, pain/tenderness of skin N05, N06, S01 |
|  | Cough, sputum R05, R25 |  | Pruritus (without xerosis/dry skin) S02 |
|  | Desquamation S21 |  | Rash, papule, lump, swelling (localized/generalized) S04-S07 |
|  | Diarrhoea D11 |  | Symptom or complaint: |
|  | Dizziness, vertigo, presyncope N17 |  | eye, eyelid F01-F29 |
|  | Dyspnoea, shortness of breath, wheezing R02, R03, R04 |  | genital, breast (female) X01-X29 |
|  | Feeling anxious/nervous/tense/angry P01, P04 |  | genital, breast, prostate, prostatism (male) Y01-Y29 |
|  | Feeling depressed P03 |  | hair/scalp S23, S24 |
|  | Feeling ill A05 |  | knee (pain, etc) L15 |
|  | Fever, chill A02, A03 |  | mouth/tongue/lip/Jaw D20, L07 |
|  | Haematemesis, vomiting blood D14 |  | nail S22 |
|  | Haemoptysis, expectoration of blood R24 |  | nose (epistaxis, etc) R06, R07, R08 |
|  | Insomnia, somnolence, sleep disturbance P06 |  | shoulder (pain, etc) L08 |
|  | Melaena, rectal bleeding D15, D16 |  | teeth/gum D19 |
|  | Nausea, vomiting D09, D10 |  | throat (dysphagia, throat pain, etc) D21, R21 |
|  | Pain |  | urinary (dysuria, haematuria, etc) U01-U29 |
|  | Abdominal/flank pain (except dyspepsia, epigastric pain, heartburn) D01, D02, D06, L05 |  | Skin colour change, depigmentation, hyperpigmentation S08 |
|  | Arthralgia, myalgia, muscle/joint pain L18, L20 |  | Sweating problem, perspiration A09 |
|  | Arthralgia, myalgia, muscle/joint pain L18, L20 |  | Swollen leg, peripheral oedema K07 |
|  | Chest pain A11, L04, R01 |  | Tremor, abnormal involuntary movement N08 |
|  | Dyspepsia, epigastric pain, heartburn D02, D03, D07 |  | Weight loss T08 |
|  | Headache N01 |  |  |
| **B) Infectious diseases** | | | |
|  | Acute bronchitis, pneumonia R78, R81 |  | Skin infection, other (cellulitis, etc) S09, S10, S76 |
|  | Conjunctivitis (except allergic) F70 |  | Tuberculosis, active A70 |
|  | Cystitis, pyelonephritis U70, U71 |  | Current |
|  | Folliculitis, furuncle, paronychia S09, S10 |  | Past |
|  | Gastroenteritis D70, D73 |  | Tuberculosis, latent A70 |
|  | Hepatitis B, HBV D72 |  | Current |
|  | Current |  | Past |
|  | Past |  | Upper respiratory infection, common cold, pharyngitis, tonsillitis, sinusitis R74, R75, R76, R80 |
|  | Hepatitis C, HCV D72 |  | Urethritis, prostatitis, STI (except vaginitis, vulvitis, balanitis), without identification of pathogen A78, U72, X74, X85, Y73, Y74 |
|  | Current |  | Urethritis, prostatitis, STI (except vaginitis, vulvitis, balanitis), with identification of pathogen |
|  | Past |  | Chlamydia X92, Y99 |
|  | HIV, AIDS B90 |  | Gonococcus X71, Y71 |
|  | Intestinal parasite D96 |  | Herpes X90, Y72 |
|  | Mycosis, fungal infection (except genital and pityriasis versicolor) A78, S74, S75 |  | Other pathogen (except HIV, HBV, HCV) |
|  | Otitis (externa or media) H70-H74 |  | Syphilis X70, Y70 |
|  | Pityriasis versicolor S74 |  | Vaginitis, vulvitis, balanitis X72, X73, X84, Y75 |
|  | Scabies, pediculosis S72, S73 |  | Warts S03 |

| **C) General internal medicine (without injuries)** | | | |
| --- | --- | --- | --- |
|  |  |  |  |
|  | Abdominal hernia (inguinal, etc) D89, D91 |  | Haemorrhoids K96 |
|  | Abnormal white cells (leucopenia, etc) B84 |  | Hiatus hernia D90 |
|  | Acne S96 |  | Hunger strike P29 |
|  | Allergic rhino/conjunctivitis R97, F71 |  | Hypertension, high blood pressure K85, K86, K87 |
|  | Allergy, food allergy/intolerance A92, D99 |  | Hypotension, orthostatic hypotension K29, K88 |
|  | Anaemia B78-B82 |  | Hypothyroidism T86 |
|  | Benign prostatic hypertrophy Y85Asthma R96 |  | Irritable bowel syndrome D93 |
|  | Cholecysitis, cholelithiasis, biliary colic D98 |  | Ischaemic heart disease K74, K75, K76 |
|  | Death A96 |  | Migraine, tension headache N89, N95 |
|  | Diabetes T89, T90 |  | Absence of disease A97 |
|  | Disease: |  | Obesity (BMI >=30) T82 |
|  | ear/mastoid (except otitis) H76-H99 |  | Osteoarticular chest pain L04 |
|  | eye/admix (except conjunctivitis) F72-F99 |  | Phlebitis, venous thrombosis K94 |
|  | liver (except HBV, HCV) D97 |  | Pregnancy, delivery, lactation W |
|  | mouth/tongue/lip (except upper respiratory infection, common cold, pharyngitis, tonsillitis, sinusitis) D83 |  | Pruritis with xerosis/dry skin S21 |
|  | allergic rhino/conjunctivitis R97, F71 |  |  |
|  | nose (except upper respiratory infection, common cold, pharyngitis, tonsillitis, sinusitis) R99 |  | Renal failure U99 |
|  | oesophagus (except hiatus hernia) D84 |  | Seborrhoeic dermatitis, dandruff S86 |
|  | skin, other S99 |  | Urinary calculus/lithiasis U95 |
|  | teeth/gum D82 |  | Urticaria S98 |
|  | Dyslipidaemia, hypercholesterolaemia T93 |  | Valvular heart disease, heart murmur K81, K83 |
|  | Eczema, contact dermatitis S87, S88 |  | Varicose veins of the leg, venous insufficiency of the leg K95 |
|  | Gastritis, duodenitis, duodenal/gastric ulcer D85, D86, D87 |  | Vitamin/nutritional deficiency (without anaemia) T91 |
| **D) Injuries and rheumatology (except deliberate self-harm)** | | | |
|  | Alleged victim of violence (by police or guard) L62, Z25 |  | Injury eye (haemorrhage, etc) F75, F76, F79 |
|  | Animal/human bite S13 |  | Osteoarthritis L89, L90, L91 |
|  | Contusion/ecchymosis/bruising, without skin wound S16 |  | Post traumatic muscle injury (muscle tear, etc) L18, L19 |
|  | Contusion/ecchymosis/bruising, with skin wound (cut, laceration, etc) S17, S18 |  | Shoulder syndrome, rotator cuff tear L92 |
|  | Dislocation, subluxation L80 |  | Sprain/strain |
|  | Fracture |  | Ankle L77 |
|  | Head L76 |  | Knee L78 |
|  | Lower limb (femur and distal) L76 |  | Other joint L79 |
|  | Spine (cervical, dorsal, lumbar), sacrum L76 |  | Wrist L79 |
|  | Thorax (sternum, rib, clavicle, scapula) L76 |  | Tendinitis, tendinopathy, bursitis, peri-articular injury (except shoulder syndrome, rotator cuff tear, injury knee other) L87 |
|  | Upper limb (humerus and distal) L76 |  | Tennis elbow, epicondylitis L93 |
|  | Injury knee, other (meniscus, etc) L96, L99 |  | Traumatic injury/trauma, other A80 |
| **E) Toxicology and self aggressive behavior (deliberate self-harm)** | | | |
|  | Benzodiazepine use P1 |  | Intentional ingestion of foreign body, hanging D79, P77 |
|  | Cannabis/hashish P19 |  | Medication abuse, self poisoning A84, P18, P77 |
|  | Cocaine P19 Active / Previous |  | Self-mutilation, scarification P77, S18 |
|  | Excessive alcohol use P15 |  | Tobacco abuse P17 |
|  | Heroin P19 Active / Previous |  |  |
| **F) Psychiatry** | | | |
|  | Adjustment disorder P02 |  | Post traumatic stress disorder, PTSD P82 |
|  | Bipolar disorder P73 |  | Psychiatric disorder, other P99 |
|  | Depression, depressive disorder P76 |  | Psychosis, schizophrenia P71, P72, P98 |
|  | Personality disorder P80 |  |  |
|  |  |  |  |
